# Reversible Resistance Induced by FLT3 Inhibition: A Novel Resistance Mechanism in Mutant FLT3-Expressing Cells

**DOI:** 10.1371/journal.pone.0025351

**Published:** 2011-09-28

**Authors:** Ellen Weisberg, Arghya Ray, Erik Nelson, Sophia Adamia, Rosemary Barrett, Martin Sattler, Chengsheng Zhang, John F. Daley, David Frank, Edward Fox, James D. Griffin

**Affiliations:** 1 Department of Medical Oncology/Hematologic Neoplasia, Dana Farber Cancer Institute, Boston, Massachusetts, United States of America; 2 Cytogenetic Core Facility, Dana Farber Cancer Institute, Boston, Massachusetts, United States of America; 3 Hematologic Neoplasia Flow Cytometry Facility, Dana Farber Cancer Institute, Boston, Massachusetts, United States of America; 4 Molecular Diagnostics Laboratory, Dana Farber Cancer Institute, Boston, Massachusetts, United States of America; Roswell Park Cancer Institute, United States of America

## Abstract

**Objectives:**

Clinical responses achieved with FLT3 kinase inhibitors in acute myeloid leukemia (AML) are typically transient and partial. Thus, there is a need for identification of molecular mechanisms of clinical resistance to these drugs. In response, we characterized MOLM13 AML cell lines made resistant to two structurally-independent FLT3 inhibitors.

**Methods:**

MOLM13 cells were made drug resistant via prolonged exposure to midostaurin and HG-7-85-01, respectively. Cell proliferation was determined by Trypan blue exclusion. Protein expression was assessed by immunoblotting, immunoprecipitation, and flow cytometry. Cycloheximide was used to determine protein half-life. RT-PCR was performed to determine FLT3 mRNA levels, and FISH analysis was performed to determine FLT3 gene expression.

**Results and Conclusions:**

We found that MOLM13 cells readily developed cross-resistance when exposed to either midostaurin or HG-7-85-01. Resistance in both lines was associated with dramatically elevated levels of cell surface FLT3 and elevated levels of phosphor-MAPK, but not phospho-STAT5. The increase in FLT3-ITD expression was at least in part due to reduced turnover of the receptor, with prolonged half-life. Importantly, the drug-resistant phenotype could be rapidly reversed upon withdrawal of either inhibitor. Consistent with this phenotype, no significant evidence of FLT3 gene amplification, kinase domain mutations, or elevated levels of mRNA was observed, suggesting that protein turnover may be part of an auto-regulatory pathway initiated by FLT3 kinase activity. Interestingly, FLT3 inhibitor resistance also correlated with resistance to cytosine arabinoside. Over-expression of FLT3 protein in response to kinase inhibitors may be part of a novel mechanism that could contribute to clinical resistance.

## Introduction

A constitutively activated, mutated version of the class III receptor tyrosine kinase, FLT3 (*F*ms-*L*ike *T*yrosine kinase-3; STK-1, human *S*tem Cell *T*yrosine *K*inase-1; or FLK-2, *F*etal *L*iver *K*inase-2), is expressed in approximately 30% of AML patients and a subset of ALL patients [1]. The most prevalent form of mutant FLT3, present in approximately 20–25% of AML patients and less than 5% of myelodysplastic syndrome (MDS) patients, occurs as internal tandem duplications (ITD) within the juxtamembrane domain [2]–[6]. Gain-of-function point mutants in FLT3, typically in the kinase activation loop (often at position 835) [7] are detected in about 7% of patients with AML. Additional FLT3 mutations that have been identified include N841I [8], Y842C [9], and novel, weakly activating point mutations in a 16 amino acid stretch of the FLT3 juxtamembrane [10].

Thus, mutant FLT3 represents an attractive target for the therapy of AML. Several FLT3 inhibitors, including the N-indolocarbazole, PKC412 (midostaurin; N-benzoyl-staurosporine, Novartis Pharma AG) [11], have shown sufficient efficacy and safety profiles to warrant further study in combination with standard therapies in advanced clinical trials. However, inhibitors of FLT3 have generally elicited partial and transient clinical responses in early trials [12], [13]. The observed suboptimal clinical responses, coupled with detection of drug-resistant leukemic blast cells in FLT3 inhibitor-treated AML patients, have made understanding clinical resistance to FLT3 inhibitors a priority. The cause for clinical resistance has not yet been identified and there is little evidence for FLT3 mutations that are analogous to the BCR-ABL gate-keeper mutations in CML, observed in response to imatinib and related compounds.

In a phase II trial of the FLT3 inhibitor, lestaurtinib (CEP701), which tested the agent in older AML patients (both mutant FLT3- and wild-type FLT3-expressing) that were not considered fit for intensive chemotherapy, the majority of clinical responses observed were only of short duration (25 days was the median time to disease progression) [14]. Interestingly, in this study, most (13/14) of patients in which blast surface FLT3 expression was able to be measured showed an elevation in blast surface FLT3 expression while treatment with lestaurtinib ensued. Authors of this study speculated that this up-regulation of FLT3 receptor expression might have contributed to the limited clinical benefit of dose escalation from 60 mg to 80 mg, and thus might represent a potential mechanism of drug resistance.

Several recent cell line-based studies have attempted to investigate possible underlying mechanism(s) of FLT3 inhibitor resistance, using mutant FLT3-expressing cell lines exposed for prolonged periods of time to increasing concentrations of various inhibitors [15]–[17]. Culture of the MV4-11 cell line (which has FLT3-ITD in both alleles) over a span of several months in the presence of increasing concentrations of the FLT3 inhibitor, ABT-869, led to an ABT-869-resistant line characterized by activation of STAT pathways and over-expression of the IAP, survivin [16], [18], [19]. Similar long-term exposure of MV4-11 cells to PKC412 led to a PKC412-resistant line characterized by clonal alterations at Chromosome 13q, and DNA-oligonucleotide microarray analysis and RT-PCR revealed modest changes in levels of Mcl-1 and FLT3 ligand, although a sizable up-regulation of the Notch signaling ligand, JAG1 [17]. Ras mutations were found in another assay system that utilized MOLM14 cells (an AML-M5 line having one wt FLT3 allele and one FLT3-ITD allele) made resistant to FLT3 inhibitors, CEP-5214 [20] and CEP-701 [21] following long-term exposure to these agents [15].

Thus, there are a number of potentially significant contributors to FLT3 inhibitor resistance, generally characterized by persistent activation of signaling through unknown mechanisms, activation of survival pathways, or potentially activation of bypass pathways, such as Ras. It is not yet clear whether these various mechanisms of resistance are specific to each inhibitor or each cell line.

Here we have used two structurally distinct FLT3 kinase inhibitors, PKC412 [11], and the novel type II ATP competitive inhibitor, HG-7-85-01 [22], to generate resistant cell lines using the human AML cell line, MOLM-13. The cell lines obtained were cross-resistant, and in both cases, resistance was associated with a striking over-expression of the mutant FLT3 receptor, a finding reflective of the previously observed up-regulation of surface FLT3 receptor expression in FLT3 inhibitor-treated patients [14]. The mechanism and effects of this type of tyrosine kinase inhibitor resistance are described in this study.

## Materials and Methods

### Cell lines and cell culture

The human AML-derived, FLT3-ITD-expressing cell line, MOLM-13 (DSMZ (German Resource Centre for Biological Material), was engineered to express luciferase fused to neomycin phosphotransferase (pMMP-LucNeo) by transduction with a VSVG-pseudotyped retrovirus as previously described [23]. For development of PKC412-resistant MOLM13-luc+ cells (MOLM13-R-PKC412), MOLM13-luc+ cells were started in culture in the presence of 1 nM PKC412 for approximately 3 weeks. From day 22 through day 79 post-commencement of culture in the presence of inhibitor, the concentration of PKC412 was gradually increased from 1 nM to 10 nM. From day 79 through day 96, the concentration of PKC412 was gradually increased from 10 nM to 50 nM.

For development of HG-7-85-01-resistant MOLM13-luc+ cells (MOLM13-R-HG-7-85-01), MOLM13-luc+ cells were started in culture in the presence of 1 nM HG-7-85-01 for approximately 3 weeks. From day 22 through day 79 post-commencement of culture in the presence of inhibitor, the concentration of HG-7-85-01 was gradually increased from 1 nM to 5 nM. From day 79 through day 96, the concentration of HG-7-85-01 was either maintained at 5 nM or was gradually increased to 10 nM. Development of PKC412-resistant cells derived from drug-resistant colonies (MOLM13-R-PKC412 (CFU)) is described in the supplementary data section.

Cell lines were cultured with 5% CO_2_ at 37°C in RPMI (Mediatech, Inc., Herndon, VA) with 10% fetal calf serum (FCS) and supplemented with 1% L-glutamine. Culture media for PKC412-resistant MOLM13-luc+ cells derived from either long-term culture or drug-resistant colonies was supplemented with a final concentration of 50 nM PKC412. Culture media for HG-7-85-01-resistant MOLM13-luc+ cells was supplemented with a final concentration of 5 nM or 10 nM HG-7-85-01, as indicated for each study.

### Chemical compounds and biologic reagents

HG-7-85-01 was synthesized in the laboratory of Dr. Nathanael Gray, DFCI, Boston, MA. PKC412 was synthesized by Novartis Pharma AG, Basel, Switzerland. Compounds were initially dissolved in DMSO to make 10 mM stock solutions, and then were serially diluted to obtain final concentrations for *in vitro* experiments. Cycloheximide (Sigma, St Louis, MO) was prepared as a stock solution (10 mg/mL).

### Antibodies and immunoblotting

All antibodies used for immunoblotting were diluted at 1∶1000. Anti-p-Tyr (clone 4G10) was purchased from Upstate Biotechnology (Lake Placid, NY). Purchased from Santa Cruz Biotechnology (Santa Cruz, CA) were anti-FLT3/Flk-2 (C-20) (rabbit, sc-479), phospho-STAT3 (rabbit, #9131), total STAT3 (C-20) (rabbit, Sc-482). Bcl-2 (Cat # 1017-1, rabbit mAb) was purchased from EPITOMICS (Burlingame, CA). DR-5 (ab47179, rabbit) was purchased from AbCam. Antibodies purchased from Cell Signaling Technology (Danvers, MA) included phospho-MAPK-p44/42 (T202/Y204) (rabbit, #9101S), total MAPK (rabbit, #9102), p-MEK1/2 (S217/221) (41G9) (rabbit, #9154S), total MEK1/2 mAb (47E6) (rabbit, #9126), phospho-STAT5 Tyr694 (rabbit, #9351S), total STAT5 (3H7) (rabbit, #9358 mAb), P27 Kip1 (rabbit, #2552), Mcl-1 (D35A5) (rabbit mAb #5453), Bax (D2E11) mAb (#5023), BAK (rabbit, #3814S), BIM (rabbit, #2819), and BID (7A3) (mouse Ab).

Loading controls included alpha-tubulin (Sigma, St Louis, MO) (1∶5000 dilution), beta-actin (mouse monoclonal, Sigma, St Louis, MO) (1∶1000 dilution), and GAPDH (Cell Signaling Technology, Danvers, MA) (1∶1000 dilution).

Protein lysate preparation and immunoblotting were carried out as previously described [11].

### Proliferation studies

Cell counts for proliferation studies were obtained using the trypan blue exclusion assay, as previously described [11]. Error bars represent the standard error of the mean for each data point.

### Flow cytometry

Drug-sensitive and drug-resistant MOLM13-luc+ cells were stained in the dark for 20–30 minutes with a CD135-PE antibody for detection of FLT3 receptor or an IgG aPE(1) antibody was used as a control for nonspecific effects (as per manufacturer's suggestions). Following the staining, FACS analysis was performed.

### RT-PCR

RNA was isolated from cells using an RNeasy kit from Qiagen (Valencia CA). cDNA was generated using the TaqMan Reverse Transcription Kit from Applied Biosystems (Foster City, CA). Quantitative polymerase chain reaction was performed in triplicate on a 7500 real-time PCR system (Applied biosystems) using SYBR Green Master Mix from Applied Biosystems using the following primers: forward, CAGATGCAGAAGAAGCGATG and reverse, TGAGCCTGCGGAGAGAGTAG. Data are expressed as mean fold change of three replicates. For the internal control: HPRT, forward, GAACGTCTTGCTCGAGATGTG and reverse, CCAGCAGGTCAGCAAAGAATT.

### FISH analysis

FISH analysis was performed to assess the copy number of FLT3 in three human cancer cell lines: MOLM13 (MOLM13-S), PKCRes+ (MOLM13-R-PKC412), and PKCRes- (MOLM13-R-PKC412, drug-deprived for >3 weeks). Briefly, the cells were harvested and treated with 0.075 M KCl for 20 min at room temperature, and subsequently fixed with 3∶1 Methanol∶ Acetic Acid. The fixed cells were hybridized in a humidified chamber with a human chromosome 13 specific reference probe (RP11-151A6, green color) and a human FLT3 probe (RP11-136G6, orange color) at 37°C for 48 hr. The slides were washed with 50% Formamide/2XSSC (pH 7.2), 2X SSC, and 1XPBD. The slides were stained with DAPI and examined with Olympus BX51 microscope for signals.

## Results

### FLT3 inhibitor-sensitive (MOLM13-S) cells and FLT3 inhibitor-resistant (MOLM13-R) cells: Comparison of proliferation in the presence of inhibitor, cellular tyrosine phosphorylation, and FLT3 autophosphorylation

**Figure 1:**
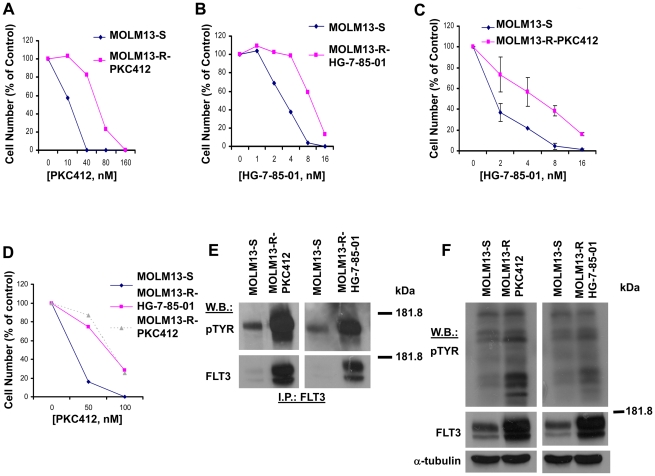
FLT3 inhibitor-sensitive and FLT3 inhibitor-resistant cells: Comparison of proliferation in the presence of inhibitor, cellular tyrosine phosphorylation, and FLT3 autophosphorylation. (A) Proliferation/drug sensitivity of drug-sensitive MOLM13-luc+ cells (MOLM13-S) as compared to MOLM13-luc+ cells exposed for 3 days to gradually increasing concentrations of PKC412 over a span of approximately three months (MOLM13-R-PCK412). Proliferation study was performed on day 97 since the start of culture of cells in the presence of PKC412. (B) Proliferation/drug sensitivity of MOLM13-S cells as compared to MOLM13-luc+ cells exposed for 3 days to gradually increasing concentrations of HG-7-85-01 over a span of approximately three months (MOLM13-R-HG-7-85-01). Proliferation study was performed on day 97 since the start of culture of cells in the presence of HG-7-85-01. (C) Approximately 2-day HG-7-85-01 treatments of MOLM13-S cells and MOLM13-R-PKC412 cells (made resistant to 50 nM PKC412 following long-term culture). (D) Approximately 3-day PKC412 treatments of MOLM13-S cells, MOLM13-R-HG-7-85-01 cells (made resistant to 5 nM HG-7-85-01 following long-term culture), and MOLM13-R-PKC412 cells (made resistant to 50 nM PKC412 following long-term culture). (E) Phospho-FLT3 and total FLT3 protein expression in drug-sensitive and drug-resistant cells. Protein lysates were prepared from MOLM13-R-PKC412 cells (resistant to 50 nM PKC412), MOLM13-R-HG-7-85-01 cells (resistant to 10 nM HG-7-85-01), and MOLM13-S cells, and were analyzed via immunoprecipitation with FLT3 and immunoblotting with antibodies to FLT3 and pTYR. (F) Total cellular tyrosine phosphorylation and total FLT3 protein expression in drug-sensitive and drug-resistant cells. Protein lysates were prepared from MOLM13-R-PKC412 cells(resistant to 50 nM PKC412), MOLM13-R-HG-7-85-01 cells (resistant to 5 nM HG-7-85-01), and MOLM13-S cells, and were analyzed via immunoblotting with antibodies to FLT3, pTYR, and alpha-tubulin as a loading control.

MOLM13-luc+ cells were grown in the presence of gradually increasing concentrations of PKC412 and HG-7-85-01, respectively, over a span of approximately three months. Cells made resistant to PKC412 (MOLM13-R-PKC412) (average IC50 derived from several experiments of approximately 55 nM) were several fold less sensitive to PKC412 than drug-sensitive cells (MOLM13-S) (average IC50 derived from several experiments of approximately 11 nM) (Figure 1A). Similarly, the sensitivity of cells made resistant to HG-7-85-01 (MOLM13-R-PKC412) (average IC50 derived from several experiments of approximately 8 nM) was several fold lower than that of MOLM13-S cells (average IC50 derived from several experiments of approximately 2 nM) (Figure 1B). In addition, MOLM13-R-PKC412 cells showed less sensitivity to HG-7-85-01 than MOLM13-S cells (Figure 1C), and MOLM13-R-HG-7-85-01 cells showed sensitivity to PKC412 that was comparable to that shown by MOLM13-R-PKC412 (Figure 1D). These results suggest cross-resistance of MOLM13-R-HG-7-85-01 and MOLM13-R-PKC412, which may be due to their shared over-expression of mutant FLT3.

Levels of phospho-FLT3, total cellular tyrosine phosphorylation, and total FLT3 are higher in MOLM13-R-PKC412 and MOLM13-R-HG-7-85-01 cells than in MOLM13-S cells (Figure 1E–F). A parallel study of PKC412-resistant cells derived from pooled colonies growing in methylcellulose in the presence of 100 nM PKC412 (MOLM13-R-PKC412-CFU) similarly showed elevated phospho-FLT3 and total FLT3 protein levels, as compared to MOLM13-S cells (Figure S1).

### MOLM13-S cells and MOLM13-R cells: Comparison of FLT3 kinase activity and tyrosine phosphorylation of signaling molecules associated with FLT3 signaling

**Figure 2:**
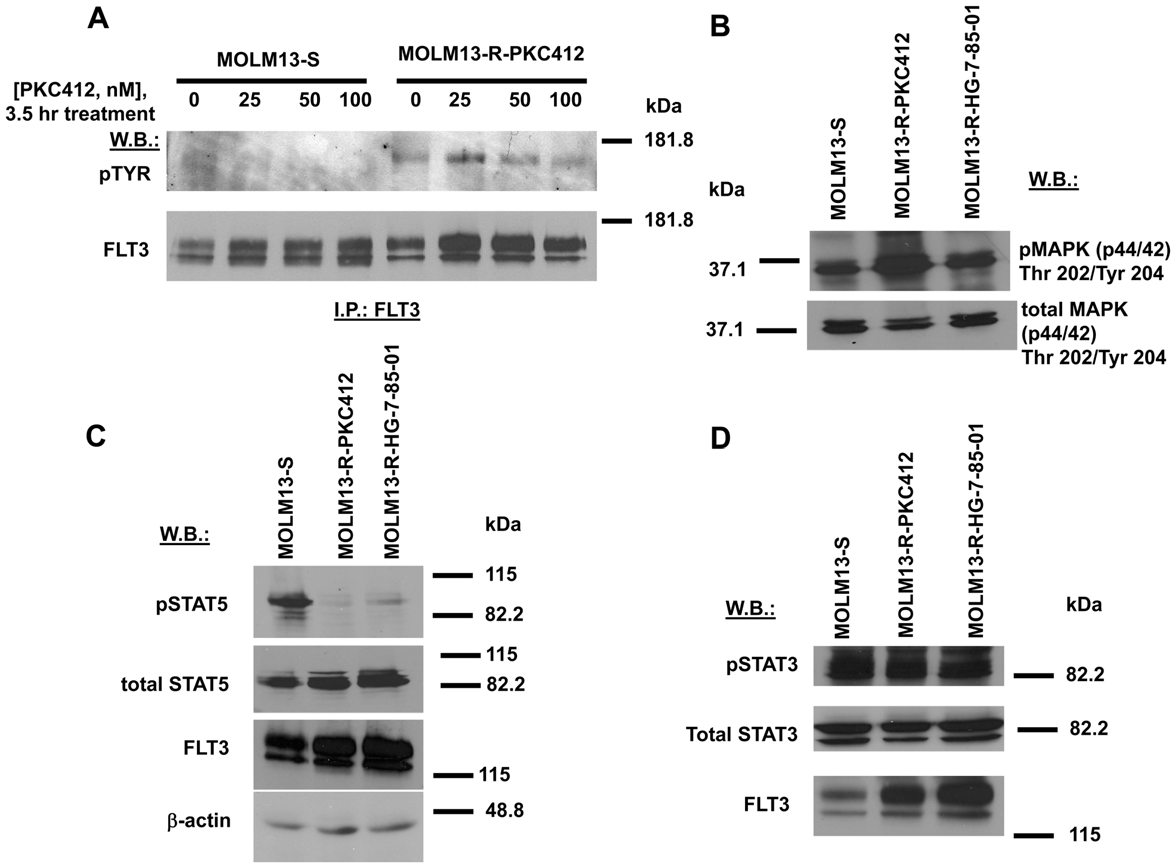
FLT3 inhibitor-sensitive and FLT3 inhibitor-resistant cells: Comparison of FLT3 kinase activity and tyrosine phosphorylation of signaling molecules associated with FLT3 signaling. (A) Effects of PKC412 inhibition of phospho-FLT3 in MOLM13-S and MOLM13-R-PKC412 cells. (B) Phospho-MAPK (p44/42) expression in MOLM13-S and drug-resistant cells as measured by immunoblot. (C–D) Phospho-STAT expression in MOLM13-S and drug-resistant cells as measured by immunoblot. For A–D, PKC412-resistant MOLM13 cells resistant to 50 nM PKC412 were used, and HG-7-85-01-resistant MOLM13 cells resistant to 10 nM HG-7-85-01 were used.

FLT3 kinase activity in MOLM13-S cells was more potently inhibited by PKC412 (near-to-complete inhibition was observed at 25 nM PKC412) than FLT3 kinase activity in drug-resistant cells (FLT3 kinase activity at 100 nM was comparable to that observed in untreated cells) (Figure 2A). These results are consistent with the higher sensitivity observed for MOLM13-S cells to the inhibitory effects of PKC412 on cell proliferation, as compared to drug-resistant cells.

As activation of and crosstalk between the RAS/Raf/MEK/ERK and Jak/STAT signaling pathways play a role in the survival advantage of leukemic cells driven by mutant FLT3, we were interested in comparing the expression levels and activity of relevant signaling components in drug-sensitive and drug-resistant cells. Higher phospho-MAPK expression was observed in MOLM13-R-PKC412 cells grown in the continuous presence of PKC412, as compared to MOLM13-S cells (Figure 2B). Correspondingly, a modest elevation of phospho-MEK expression was observed in MOLM13-R-PKC412 cells (Figure S2). Paradoxically, despite over-expression of FLT3 levels in MOLM13-R-PKC412 and MOLM13-R-HG-7-85-01 cells grown in the presence of inhibitor, there was a striking decrease in levels of pSTAT5 in both drug resistant lines, as compared to MOLM13-S cells (Figure 2C). This finding, however, did not apply to pSTAT3, which remained unchanged in drug-resistant as compared to MOLM13-S cells (Figure 2D).

Deregulation of proteins associated with apoptotic signaling is often associated with drug resistance. As such, we decided to investigate the expression of proteins including Bcl-2, Mcl-1, Bax, BIM, BAK, and BID, in MOLM13-R-PKC412 and MOLM13-R-HG-7-85-01 cells. There was no observation of significant up-regulation of anti-apoptotic Bcl-2 family proteins or down-regulation of pro-apoptotic Bcl-2 family proteins (data not shown). We also investigated the expression of death receptor 5 (DR5), a member of the tumor necrosis factor receptor (TNFR) family, and did not observe differences in its expression in drug-sensitive and drug-resistant cells (data not shown).

### FLT3 inhibitor-resistant cells: Drug influence on rate of proliferation

**Figure 3:**
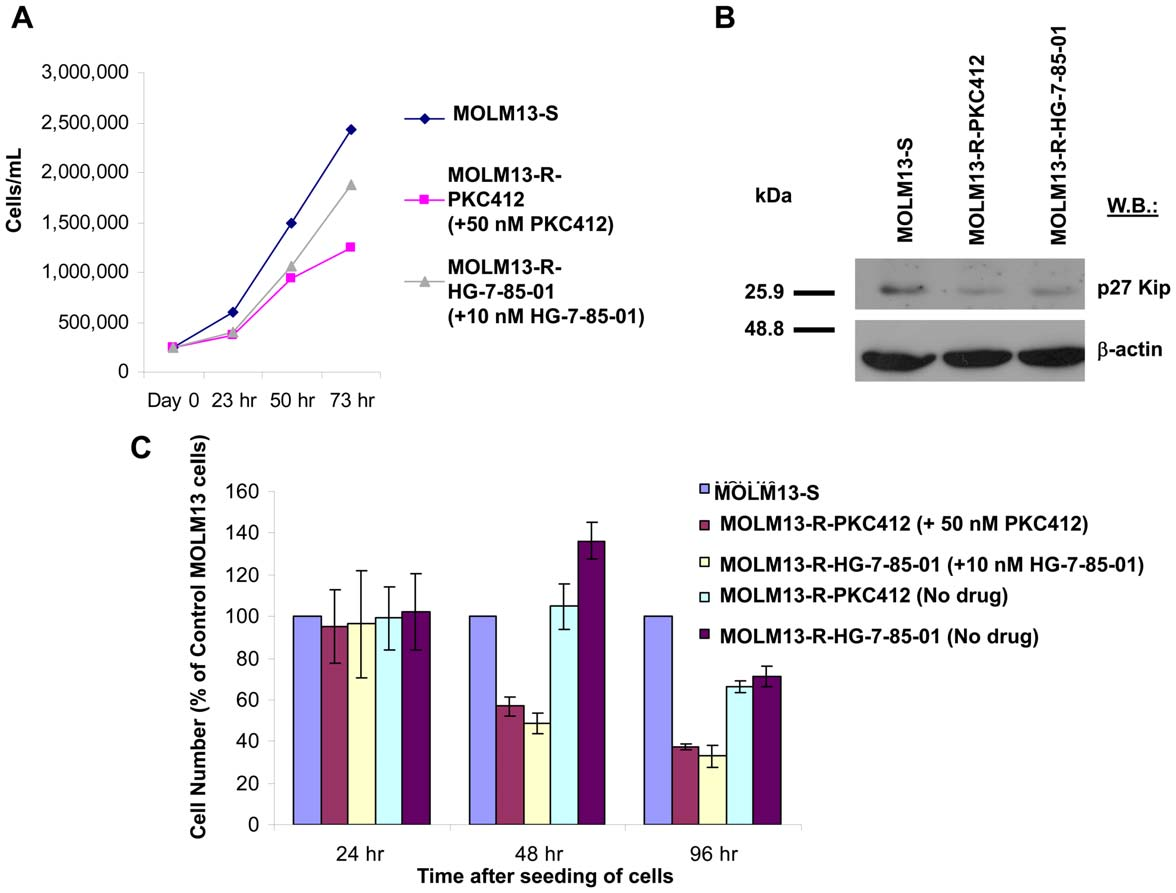
FLT3 inhibitor-resistant cells: Drug influence on rate of proliferation. (A) Comparison of rate of proliferation of MOLM13-S, MOLM13-R-PKC412 (cultured in the continuous presence of 50 nM PKC412), and MOLM13-R-HG-7-85-01 (cultured in the continuous presence of 10 nM HG-7-85-01). (B) p27 Kip1 expression in MOLM13-S, MOLM13-R-PKC412 (cultured in the continuous presence of 50 nM PKC412), and MOLM13-R-HG-7-85-01 (cultured in the continuous presence of 10 nM HG-7-85-01). (C) Cell proliferation of MOLM13-S and drug-resistant cells at various time points; drug-resistant cells were tested in both the absence and presence of inhibitor. Experiments were performed in triplicate.

The rate of growth of drug-resistant cells grown in the continuous presence of inhibitor was slower than that of MOLM13-S cells (Figure 3A). This observation was consistent with a decrease in expression levels of the cell cycle protein, p27 Kip (Figure 3B). Interestingly, however, removal of inhibitor from the culture medium of drug-resistant cells led to a higher rate of cell growth similar to that observed in MOLM13-S cells (Figure 3C).

### FLT3 inhibitor-resistant cells: Drug influence on proliferation and FLT3-associated signaling molecules

**Figure 4:**
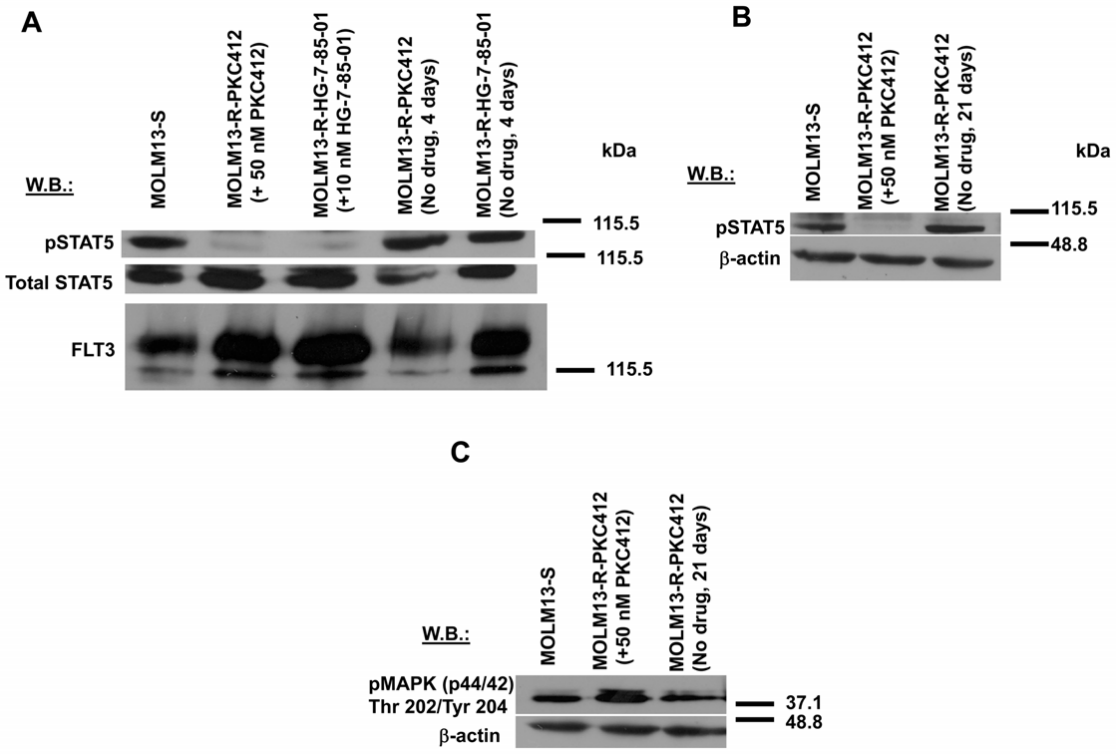
FLT3 inhibitor-resistant cells: Drug influence on FLT3-associated signaling molecules. (A) Phospho-STAT5 and FLT3 expression in drug-resistant cells cultured in the continuous presence of drug, or the absence of drug for 4 days. For this study, PKC412-resistant MOLM13 cells resistant to 50 nM PKC412 were used, and HG-7-85-01-resistant MOLM13 cells resistant to 10 nM HG-7-85-01 were used. (B) Phospho-STAT5 expression and (C) phospho-MAPK expression in PKC412-resistant cells cultured in the continuous presence of 50 nM PKC412 or its absence for 3 weeks.

Drug withdrawal for several days or weeks resulted in increased sensitivity of MOLM13-R-PKC412 and MOLM13-R-HG-7-85-01 cells to the inhibitory effects of PKC412 and HG-7-85-01, respectively, on cell proliferation (Figure S3(A–C)). Consistent with this, levels of phospho-STAT5 were increased in drug-resistant cells deprived of inhibitor for several days or several weeks to levels comparable to those observed in MOLM13-S cells (Figure 4A, B). Similarly, levels of phospho-MAPK protein in MOLM13-R cells deprived of inhibitor were comparable to baseline control levels in MOLM13-S cells (Figure 4C). In addition, withdrawal of HG-7-85-01 for six days from MOLM13-R-HG-7-85-01 led to an increase in sensitivity of the cells to PKC412, or a partial loss of cross-resistance to PKC412 (Figure S3(D)), and the withdrawal of PKC412 from MOLM13-R-PKC412 for 2, 5, and 7 days, or pulsing with 50 nM PKC412 every 2–3 days for up to 9 days led to a partial loss of cross-resistance to HG-7-85-01 (Table 1 and Figure S3 (E–I)).

**Table 1:** Fold differences in IC50 values obtained for HG-7-85-01-treated MOLM13-R-PKC412 cells cultured in the presence and absence of PKC412 prior to assay.

Cell type/condition	IC50 (fold increase)
MOLM13-S	1
MOLM13-R-PKC412	
(continuous PKC412 treatment prior to assay)	2.7
MOLM13-R-PKC412	
(condition #1)	1.5
MOLM13-R-PKC412	
(condition #2)	1.8
MOLM13-R-PKC412	
(condition #3)	0.97
MOLM13-R-PKC412	
(condition #4)	1.4

MOLM13-R-PKC412 cells were either in the continuous presence of PKC412 prior to treatment with HG-7-85-01 (PKC412 was washed out on the day of the set up of the proliferation study), or they were cultured according to the following treatment conditions: Condition #1: Two-day withdrawal of PKC412 from MOLM13-R-PKC412 cells prior to assay. Condition #2: Two days of PKC412 treatment of MOLM13-R-PKC412, two days of PKC412 withdrawal, three days of PKC412 treatment, and two days of PKC412 withdrawal prior to assay. Condition #3: Five days of PKC412 withdrawal prior to assay. Condition #4: Seven days of PKC412 withdrawal prior to assay. IC50 values are presented as fold increases over IC50 obtained for HG-7-85-01 against MOLM13-S cells.

### FLT3 inhibitor-resistant cells: Drug influence on FLT3 protein activity, stabilization and half-life

**Figure 5:**
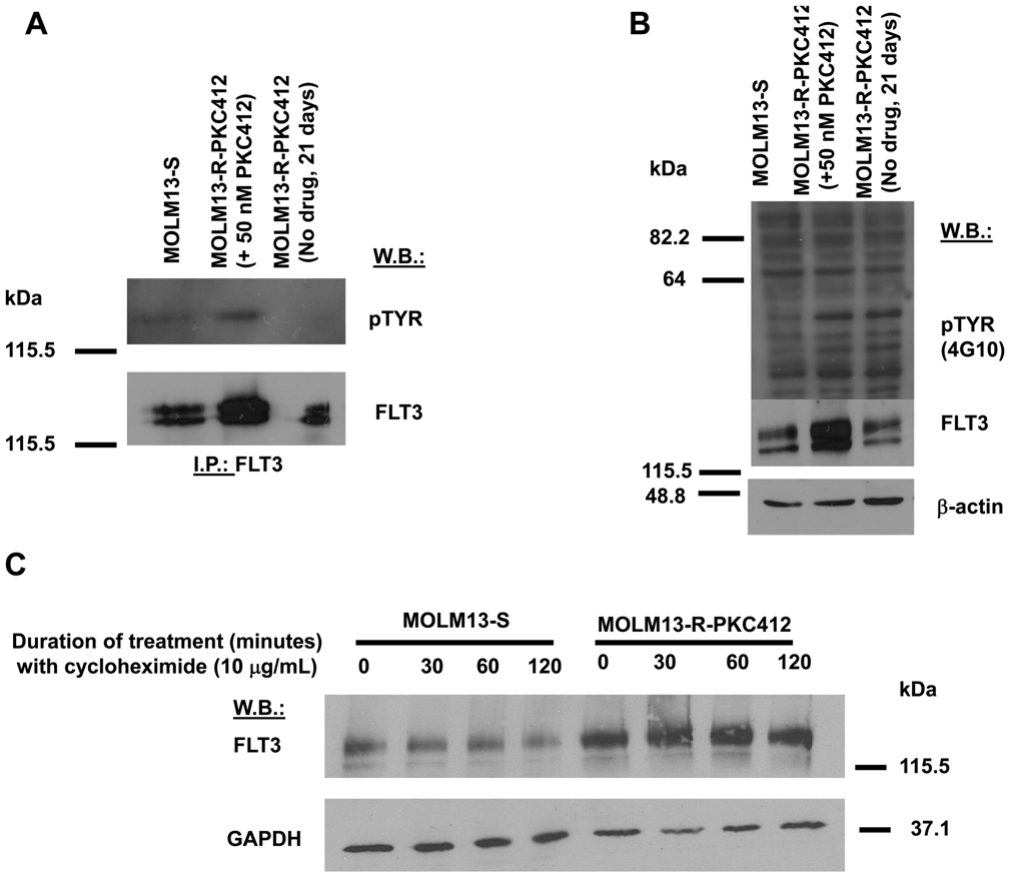
FLT3 inhibitor-resistant cells: Drug influence on FLT3 protein activity, stabilization and half-life. (A) Phospho-FLT3 and total FLT3 protein expression in MOLM13-S cells, as compared to MOLM13-R-PKC412 cells (cultured in the continuous presence of 50 nM PKC412 or its absence for 3 weeks). Protein lysates were prepared and analyzed via immunoprecipitation with FLT3 and immunoblotting with antibodies to FLT3 and pTYR. (B) Total cellular tyrosine phosphorylation and total FLT3 protein expression in MOLM13-S cells, as compared to MOLM13-R-PKC412 cells (cultured in the continuous presence of 50 nM PKC412 or its absence for 3 weeks). Protein lysates were prepared and analyzed via immunoblotting with antibodies to FLT3, pTYR, and beta-actin as a loading control. (C) Cycloheximide (10 µg/mL) treatment of MOLM13-S cells and MOLM13-R-PKC412 cells for the indicated times.

Expression levels of phospho-FLT3 and total FLT3 in drug-resistant cells deprived of drug for several weeks were comparable to those observed in MOLM13-S cells (Figure 5A). Drug-resistant cells deprived of inhibitor for several weeks, however, continued to show a modest elevation in total cellular tyrosine phosphorylation as compared to MOLM13-S cells, despite a reduction in FLT3 protein levels to those observed in wt control cells (Figure 5B). These results, coupled with the fact that prolonged drug withdrawal from resistant cells did not result in complete reversion to the drug sensitivity characteristic of wild-type cells, suggest that the absence of inhibitor in the culture media significantly- but not completely- abrogates the drug resistant phenotype.

We were interested in investigating the mechanism underlying the elevated FLT3 expression in drug-resistant cells cultured in the continuous presence of inhibitor. Drug-sensitive cells treated for varying periods of time with the protein synthesis inhibitor, cycloheximide, showed a time-dependent reduction in FLT3 expression, whereas elevated FLT3 expression in drug-resistant cells appeared to be unaffected by comparable cycloheximide treatment (Figure 5C). These results suggest higher stability of FLT3 in drug-resistant cells as measured by a longer protein half-life.

### Surface expression of FLT3 receptor in FLT3 inhibitor-sensitive and FLT3 inhibitor-resistant cells

**Figure 6:**
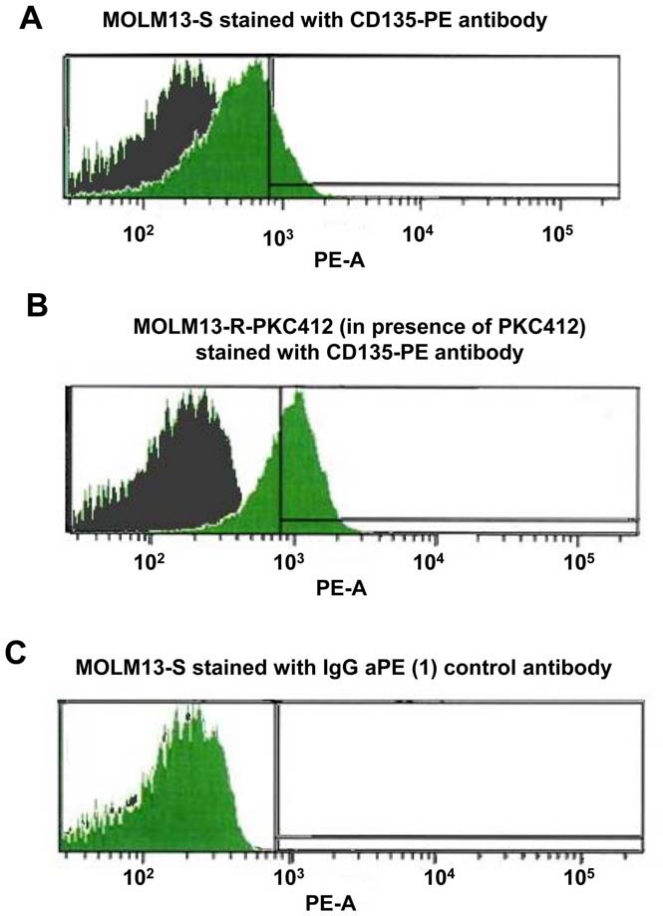
Surface expression of FLT3 receptor in FLT3 inhibitor-sensitive and FLT3 inhibitor-resistant cells. Flow cytometry was performed using a CD-135-PE antibody for detection of FLT3 receptor surface expression in MOLM13-S cells (A) versus MOLM13-R-PKC412 cells (cultured in the continuous presence of 50 nM PKC412). (B) An IgG aPE (1) antibody was used as a control (shown in C). For A: No staining MOLM13-S control (PE-A median: 126); MOLM13-S stained with CD135-PE antibody (PE-A median: 497). For B: No staining MOLM13-R-PKC412 control (in presence of PKC412) (PE-A median: 134); MOLM13-R-PKC412 (in presence of PKC412) stained with CD135-PE antibody (PE-A median: 951). For C: No staining MOLM13-S control (PE-A median: 129); MOLM13-S stained with IgG aPE (1) control antibody (PE-A median: 156). Shaded = background.

Flow cytometric analysis of FLT3 expression revealed substantially higher FLT3 expression in the drug resistant cells cultured in the continuous presence of inhibitor as compared to MOLM13-S cells (Figure 6). Thus, the elevation in FLT3 receptor expression in resistant cells appears to be predominantly due to increased surface expression of FLT3. Drug resistant cells cultured in the absence of inhibitor for several weeks showed FLT3 expression levels similar to those observed in MOLM13-S cells (Figure S4). This was again in contrast to the considerably higher surface expression of FLT3 protein observed in resistant cells cultured in the continuous presence of inhibitor (Figure S4).

### FLT3 mRNA and gene expression in FLT3 inhibitor-sensitive and FLT3 inhibitor-resistant cells

**Figure 7:**
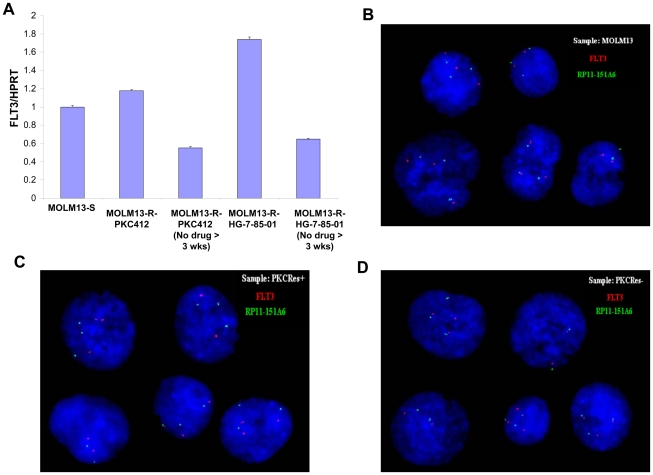
FLT3 mRNA and gene expression in FLT3 inhibitor-sensitive and FLT3 inhibitor-resistant cells. (A) RT-PCR was performed for comparison of FLT3 mRNA levels in MOLM13-S cells and drug-resistant cells cultured in the absence and presence of drug. This study is representative of two independent studies in which similar results were observed. (B–D) FISH analysis was performed for comparison of FLT3 gene expression in MOLM13-S cells (B) and drug-resistant cultured in the presence (C) and >3 week absence (D) of drug.

We were interested in investigating whether changes at the genetic or transcriptional level for FLT3 might contribute to the aberrant expression of FLT3 protein observed in drug resistant cells. RT-PCR revealed only a modest up-regulation of FLT3 mRNA in drug-resistant cells cultured in the continuous presence of inhibitor, with a modest down-regulation of FLT3 in drug-resistant cells cultured in the prolonged absence of inhibitor (Figure 7A). Densitometry results suggest on average less than 2-fold changes in FLT3 mRNA levels (normalized to HPRT) and changes in FLT3 protein levels ranging from approximately 2.5-5-fold in drug-sensitive versus drug-resistant cells (normalized to tubulin or beta-actin). These results suggest that while transcriptional changes might be playing a minor role in FLT3 regulation, the changes observed at the level of FLT3 protein are more substantial and likely to be playing a more significant role.

FISH analysis revealed no significant changes in FLT3 gene copy number in drug-sensitive and drug-resistant cells (Figure 7B–D). These results argue against FLT3 gene amplification as playing a significant role in elevation of FLT3 protein expression in drug-resistant cells.

As an important mechanism of resistance to FLT3 inhibitors is acquired point mutations in the molecular targets [24], [25], and resistance to PKC412 in patients has been attributed to pre-existing or acquired mutations in the FLT3 kinase domain [26], we searched for FLT3 point mutations by sequencing the FLT3 gene in drug-sensitive and drug-resistant cells, cultured both in the continuous presence of inhibitor as well as its absence. No mutations were detected in the internal tandem duplication domain or kinase domain of FLT3 in PKC412- or HG-7-85-01-resistant cells.

### Cross-resistance in drug-resistant cells to standard chemotherapy

As evidence exists in support of mutant FLT3 contributing to resistance of AML to chemotherapeutic agents, we were interested in comparing the sensitivities of MOLM-S cells and the mutant FLT3-over-expressing MOLM13-R cells to standard chemotherapy. Intriguingly, MOLM13-R-PKC412 showed substantially less sensitivity to the standard chemotherapeutic agent, Ara-c (IC50 was approximately 24.3-fold higher than that of MOLM13-S cells) (Figure S5). MOLM13-R-HG-7-85-01 and MOLM13-R-PKC412 (CFU) cells, similar to MOLM13-R-PKC412 cells, also showed less sensitivity to Ara-c (data not shown).

Withdrawal of PKC412 from the culture media for MOLM13-R-PKC412 cells for three days led to a partial reversal of cross-resistance to Ara-c (IC50 was approximately 4.3-fold higher than that of MOLM13-S cells) (Figure S5). Cross-resistance to Ara-c, in contrast, was not reversed by removal of PKC412 from culture media for 24 hours, and removal of PKC412 for 8 days led to partial reversal of Ara-c cross resistance that was more modest than that observed for the three-day drug withdrawal (Figure S5).

## Discussion

While the complexity of the pathogenesis and heterogeneity of AML is appreciated, the presence in a subset of patients of mutated FLT3, the ITD variant of which has been shown in mouse bone marrow transplantation assays to cause a rapidly lethal myeloproliferative disease in the absence of any significant block in granulocyte lineage cell differentiation [27], would suggest the availability of a key therapeutic target that contributes strongly to the AML phenotype in these patients. As mutant FLT3 likely enhances proliferation of primitive myeloid cells, one would predict that FLT3 kinase inhibition would translate into significant clinical benefit via inhibition of expansion of this cell population. However, in most clinical trials so far, while responses may be very rapid, they are typically partial and of very short duration. Thus, FLT3 inhibitors, as single agents and in combination with standard therapy, have had limited impact. Since clinical resistance occurs rapidly, there exists a pressing need to identify the underlying reason or reasons for the suboptimal clinical efficacy of FLT3 inhibition.

Causes of FLT3 inhibitor resistance, identified recently in a relatively small number of studies, have been multi-factorial and have ranged from identification of Ras mutations, to aberrant STAT signaling [16], and- through microarray analysis and RT-PCR- significant over-expression of a notch receptor ligand [17]. Different methodologies used to investigate mechanisms of FLT3 inhibitor resistance could at least partly be attributed to for the variability in reported resistance mechanisms associated with FLT3 inhibition, as technologies based on molecular biology and technologies based on biochemistry can significantly vary in terms of sensitivity. Even differences in the FLT3 mutations of the cell lines used for resistance studies might create disparity, as MV4-11 cells have FLT3-ITD in both alleles, and MOLM13 and MOLM14 cells have one wt FLT3 allele and one FLT3-ITD allele. However another possibility may be due to the fact that many well-known FLT3 inhibitors are broad spectrum/multi-targeted inhibitors, and unlike more selective agents- such as the Abl inhibitors used for therapy of CML, may be associated with more unique and unrelated modes of resistance. For instance, a screening assay employed to study resistance profiles of three FLT3 inhibitors, PKC412, SU5614, and sorafenib, showed non-overlapping mechanisms of resistance for the three inhibitors [28]. This is in contrast to the overlapping resistance profiles displayed for ABL inhibitors, such as imatinib, nilotinib, and dasatinib, which all show resistance to the T315I gatekeeper mutation [29], [30].

There were some similarities observed between the resistance phenotype described in our studies and those reported by others. For instance, Piloto et al. reported activation of the Ras/MEK/MAPK signaling pathway in association with CEP-5214 and CEP-701 resistance, and we similarly observed elevated phospho-ERK1/ERK2 and phospho-MEK in MOLM13-R-PKC412 cells, consistent with elevated FLT3 protein levels. Zhou et al., in contrast, reported no over-expression of phospho-ERK1/ERK2 in ABT-869-resistant cells, which is a finding consistent with our observed lack of up-regulation of phospho-ERK1/ERK2 in MOLM13-R-HG-7-85-01 cells. Zhou et al. reported cross-resistance of FLT3 inhibitor-resistant lines to structurally unrelated inhibitors, an observation we made with MOLM13-R-PKC412 and MOLM13-R-HG-7-85-01 cells, as well. Finally, no mutations in the FLT3 kinase domain or ITD were found in MOLM13-R-PKC412 and MOLM13-R-HG-7-85-01 cells, consistent with reports by Zhou et al. and Piloto et al. of no mutations being detected in the kinase domain of drug-resistant cells. Similarly, Stolzel et al. reported no mutations in the entire coding region of FLT3 in resistant cells.

Our findings suggest significant over-expression of membrane surface-bound FLT3 receptor in FLT3 inhibitor-resistant cells, which appears to be due to drug-dependent stabilization and FLT3 half-life prolongation. This is in contrast to findings reported in Zhou et al., in which immunoblotting showed similar levels of FLT3 and phospho-FLT3 in drug-sensitive and –resistant cells. However, in our studies, RT-PCR showed only modest changes in FLT3 receptor levels between drug-sensitive and –resistant cells, and FISH analysis revealed no evidence for FLT3 gene amplification. These findings are consistent with those of Stolzel et al., in which microarray data and quantitative PCR did not reveal differential FLT3 expression between wt and drug-resistant cells.

Levels of phospho-STAT5, however not total STAT5, in our study were found to be dramatically suppressed in MOLM13-R-PKC412 and MOLM13-R-HG-7-85-01 cells cultured in the continuous presence of inhibitor, whereas levels of phospho-STAT3 and total STAT3 were unchanged. We hypothesize that the reduction in phospho-STAT5 in resistant cells might be a drug-dependent compensatory homeostatic mechanism, as resistant cells over-expressing FLT3 and characterized by elevated phospho-ERK1/ERK2 and phospho-MEK may not be able to tolerate overactive FLT3 signaling. Indeed, when FLT3 inhibitor is removed from the culture media, levels of phospho-STAT5 return to normal and levels of FLT3 protein decrease to the baseline levels observed in drug-sensitive control cells. Zhou et al., in contrast, reported over-expression of phospho-STAT5 in ABT-869-resistant cells (as measured by immunoblotting), as well as other members of the STAT family. In the case of ABT-869-resistant cells, it is possible that there would be no similar need for a compensatory homeostatic mechanism characterized by down-regulation of phospho-STAT5, as ABT-869-resistant cells, unlike the drug-resistant cell lines described here, show normal FLT3 protein and phospho-ERK1/ERK2 expression levels. The presence or absence of inhibitor in the culture media is also a variable that needs to be taken into consideration when measurement of the expression or activity of drug-responsive signaling pathway components, as resistant cells in the Zhou et al. study were grown in normal medium without ABT-869 for at least 48 hours before experiments.

The over-expression of FLT3 observed in two cell lines made resistant to two structurally-unrelated FLT3 inhibitors raises the notion that FLT3 inhibitor exposure may impede the efficacy of these inhibitors by increasing levels of the target FLT3 protein- hence a possible mechanism contributing to their lack of efficacy in the clinic. Our data correlate with other findings, for instance the transient inhibition in Phase I trials of FLT3 in KW-2449-treated patients to less than 20% of baseline [31]. It has also been shown that, in at least some primary AML cells, there is continued phosphorylation of ERK, STAT5, or AKT following inhibition of FLT3-ITD, which may contribute to the limited efficacy of FLT3 inhibitors used as single agents for the treatment of mutant FLT3-positive AML [32]. In addition, it has recently been shown that FLT3-ITD inhibition of autophosphorylation does not always result in cell death, which raises the question of the importance of FLT3 signaling for some mutant FLT3-positive AML [33]. Indeed, there are alternative signaling pathways that have been shown to be important in causing drug resistance, such as aberrant, constitutively active PI3K/Akt-mediated signaling in AML, and over-expression of Cdc28 protein kinase regulatory subunit 1B (*CKS1B*), which leads to activation of Jak/STAT3 and MEK/ERK signaling pathways in multiple myeloma [34], [35]. It is therefore anticipated that the limited clinical efficacy of FLT3 inhibitors may be due to their inability to elicit sustained, complete responses in patients [36].

We have previously implicated FLT3-ITD protein over-expression in secondary FLT3 inhibitor resistance [11]. In clinical trials, FLT3 inhibitor treatment and myelosuppressive chemotherapy have been implicated in inducing FLT3 ligand expression and/or FLT3 cell surface expression [14], [37]. In the Knapper et al. study, clinical effects were only transient decreases in peripheral blood and marrow blasts in 60% of mutant FLT3-expressing patients and 23% of wild-type FLT3 patients. The majority (13) of 14 patients that FLT3 protein levels were able to be measured on day 0 of treatment and on at least one time point during treatment with lestaurtinib demonstrated increases in blast surface FLT3 expression during in vivo FLT3 inhibitor treatment. Authors of this study proposed the notion that this elevation in blast surface FLT3 expression could potentially play a role in drug resistance/limited FLT3 inhibitor efficacy. Alternatively, they suggested that lestaurtinib exposure simply leads to a return of inactivated cellular FLT3 to the cell membrane. In this same investigation, at least two patients (one wild-type and one mutant FLT3-expressing) showed prolonged clinical responses to lestaurtinib that peaked several weeks after drug withdrawal. Other studies have correlated over-expression of FLT3 protein with disease relapse [38], and over-expression of FLT3 transcript levels with risk of relapse, and a high percentage of bone marrow blasts and leukocyte counts [39], [40].

In non-small cell lung cancer (NSCLC), both the over-expression of epidermal growth factor receptor (EGFR) and MET have been implicated in transformation, and it has been suggested that the inhibition of EGFR can be bypassed by activated MET [41]. Similarly, it may be possible that FLT3 inhibition in AML is not sufficient as other tyrosine kinase inhibitors might take over the function of this oncoprotein.

Our findings suggest that there may be benefit to the use of sequential combination therapy as a way to enhance the efficacy of FLT3 inhibitors and override development of drug resistance. As we did not observe between MOLM13-S and MOLM13-R cells any significant differences in intrinsic or extrinsic apoptotic pathway-related proteins such as members of the Bcl-2 family or levels of DR5, it is conceivable that combined treatment using a FLT3 inhibitor and inhibitor of apoptotic signaling may be successful in overriding drug resistance. Indeed, we observed a positive combination effect between the IAP inhibitor, LCL161, and PKC412, against MOLM13-R-PKC412 cells, even when cultured in the presence of protective secreted cytokines in stromal conditioned media (Figure S6). Our data also point toward the potential use of FLT3 antibodies or other FLT3 target-specific therapies as a way to override drug resistance.

In addition, the fact that FLT3 inhibitor treatment might contribute to an increase in target protein that- over time- lowers drug sensitivity of mutant FLT3-expressing cells (while patients are still being treated, even if this might be reversible upon withdrawal of the drug) could be proposed as one possible reason why FLT3 inhibitors might not be as clinically effective as one might predict- seeing that development of clinical inhibitors of mutant FLT3 is biorationally-speaking a promising approach. Indeed, we observed an increase in PKC412 sensitivity of MOLM13-R-PKC412 deprived of PKC412 for varying lengths of time, as well as an increase in HG-7-85-01 sensitivity of MOLM13-R-HG-7-85-01 cells deprived of HG-7-85-01. We also observed a decrease in the extent of cross-resistance of MOLM13-R-PKC412 cells to HG-7-85-01 and MOLM13-R-HG-7-85-01 cells to PKC412 following removal of the FLT3 inhibitor each line was made resistant to, respectively. Thus, withdrawing the drug cells are resistant to may be a first step in regaining drug sensitivity, and introducing in tandem another drug (i.e. another FLT3 inhibitor) would potentially be a second step in killing the resistant cells as cross-resistance is reversed by removal of the first drug.

We found the observed decrease in sensitivity of both MOLM13-R-PKC412 and MOLM13-R-HG-7-85-01 cells to Ara-c interesting and a potentially important finding in terms of the limited clinical efficacy observed with combination treatment involving FLT3 inhibitors and standard chemotherapy. As there is evidence in support of mutant FLT3 itself contributing to resistance of AML to chemotherapeutic agents, we hypothesize that perhaps the decreased Ara-c sensitivity may be due to the over-expression of FLT3 common to both resistant cell populations. For instance, induced FLT3-ITD expression in myeloid cells was observed to correlate with Ara-c resistance due to decreased cellular uptake of Ara-c [42]. Drug resistance was accompanied by down-regulation of a transporter responsible for Ara-c cellular uptake, equilibrative nucleoside transporter 1 (ENT1), possibly mediated by upregulation of hypoxia inducible factor 1 alpha subunit (HIF1A) [42].

Cross-resistance to Ara-c, similar to cross-resistance to HG-7-85-01, was partially reversed by several day withdrawal of PKC412 from the culture medium of MOLM13-R-PKC412 cells. These results, taken together, may inspire clinicians to attempt to alter the schedule of treatment with certain FLT3 inhibitors, including sequential administration of more than one FLT3 inhibitor, or sequential administration of a FLT3 inhibitor and standard chemotherapy, such as Ara-c, as strategies to optimize efficacy and induce more sustained clinical responses in patients. However, as sequential administration of chemotherapy (first) followed by FLT3 inhibitors (last) has proven to be beneficial for FLT3 inhibitors such as CEP-701 [43], other FLT3 inhibitors, such as tandutinib, are able to synergize with standard chemotherapy in a sequence-independent fashion [44]. Considering the distinct mechanisms of resistance exhibited by FLT3 inhibitors studied thus far, the nature of each individual FLT3 inhibitor should therefore be taken into consideration when optimizing therapeutic strategies.

## Supporting Information

Figure S1 **PKC412-resistant cell line (resistant to 100 nM PKC412) developed from Colony Forming Units (CFUs).** (A) Colony assays: MOLM13-luc+ cells seeded. 100 cells/0.1 mL in IMDM. 900 uL Metho Cult. Total/well = 1 mL. Stem Cell Technologies “Metho Cult”. Methylcellulose cat # 4230 without cytokines. 9 days between seeding cells and counting. (B) PKC412-resistant cell line (resistant to 100 nM PKC412) developed from Colony Forming Units (CFUs) (MOLM13-R-PKC412 (CFU): pTYR levels and FLT3 protein levels. 12/10/09 FLT3 I.P./western for PKC412-resistant colony (derived from MOLM13-luc+ cells), and compared to wt MOLM13-luc+ cells in culture. For I.P./western, FLT3 Ab (1∶2000). For development of MOLM13-R-PKC412 (CFU) cells, colony assays were initially performed in which 100 MOLM13-luc+ cells/0.1 mL in IMDM were seeded +900 uL “complete” methylcellulose medium containing recombinant cytokines (contents: fetal bovine serum, rh SCF, rh GM-CSF, rh IL-3, Bovine Serum Albumin, methylcellulose in Iscove's MDM, 2-Mercaptoethanol, rh Erythropoietin, L-Glutamine) (MethoCult GFH4434, StemCell Technologies, Inc., Vancouver, BC). The plates also contained PKC412 at the indicated concentrations. The plates were incubated at 37°C in 5% CO2 for >1 week, and then myeloid and erythroid colonies (early progenitors with erythroid and myeloid components: CFU-GM, CFU-E, BFU-E, and CFU-GEMM) were counted on an inverted microscope. There was a total of nine days between seeding cells and counting and drug-resistant colony selection, pooling of colonies, and culture of colonies.
(TIF)

Figure S2 **Phospho-MEK expression in MOLM13-R-PKC412 and MOLM13-R-HG-7-85-01 cells.** Protein expression was assessed by immunoblotting.
(TIF)

Figure S3 **(A–D). Effects of FLT3 inhibitor withdrawal on proliferation of FLT3 inhibitor-resistant cells.** (A–B) Effects of short-term drug withdrawal on MOLM13-R-PKC412 and MOLM13-R-HG-7-85-01 cells. (C) Effects of over three week drug withdrawal on MOLM13-R-PKC412 cells. (D) Drug washout experiment: six-day drug withdrawal: effects on proliferation of MOLM13-R-HG-7-85-01 in the presence of PKC412. **(E–H). Effects of FLT3 inhibitor withdrawal on proliferation of FLT3 inhibitor-resistant cells.** Condition #1: Two-day withdrawal of PKC412 from MOLM13-R-PKC412 cells prior to assay. Condition #2: Two days of PKC412 treatment of MOLM13-R-PKC412, two days of PKC412 withdrawal, three days of PKC412 treatment, and two days of PKC412 withdrawal prior to assay. Condition #3: Five days of PKC412 withdrawal prior to assay. Condition #4: Seven days of PKC412 withdrawal prior to assay.
(TIF)

Figure S4 **Flow cytometry analyzing surface expression of FLT3 receptor in drug-sensitive cells versus drug-resistant cells cultured in the absence and presence of inhibitor.**
(TIF)

Figure S5 **Cross resistance of MOLM13-R-PKC412 cells to standard chemotherapy.** (A) Comparison of sensitivity to Ara-c of MOLM13-S and MOLM13-R-PKC412 cells in the continuous presence of PKC412 and following 24 hours of PKC412 withdrawal. (B) Comparison of sensitivity of Ara-c of MOLM13-S and MOLM13-R-PKC412 cells in the continuous presence of PKC412 and following 3-days of PKC412 withdrawal. (C) Comparison of sensitivity of Ara-c of MOLM13-S and MOLM13-R-PKC412 cells in the continuous presence of PKC412 and following 8-days of PKC412 withdrawal.
(TIF)

Figure S6 **Effects of combination of LCL161 and PKC412 on PKC412-resistant leukemia cells.** (A) Stromal-mediated rescue of PKC412-resistant MOLM13-S cells cultured for approximately 3 days in the presence of PKC412. (B) Approximately 3-day treatment of MOLM13-R-PKC412 (cultured in the absence of stromal conditioned media, or SCM) with PKC412, LCL161, or a combination of PKC412 and LCL161. (C) Approximately 3-day treatment of MOLM13-R-PKC412 cells (cultured in the presence of SCM) with PKC412, LCL161, or a combination of PKC412 and LCL161. This study was performed with one fixed concentration (1000 nM) of LCL161.
(TIF)
