# Supplementary figures and images for: Reversible Resistance Induced by FLT3 Inhibition: A Novel Resistance Mechanism in Mutant FLT3-Expressing Cells
Source: PLoS One. 2011 Sep 28;6(9):e25351. doi: 10.1371/journal.pone.0025351 (PMC3182213; doi:10.1371/journal.pone.0025351)

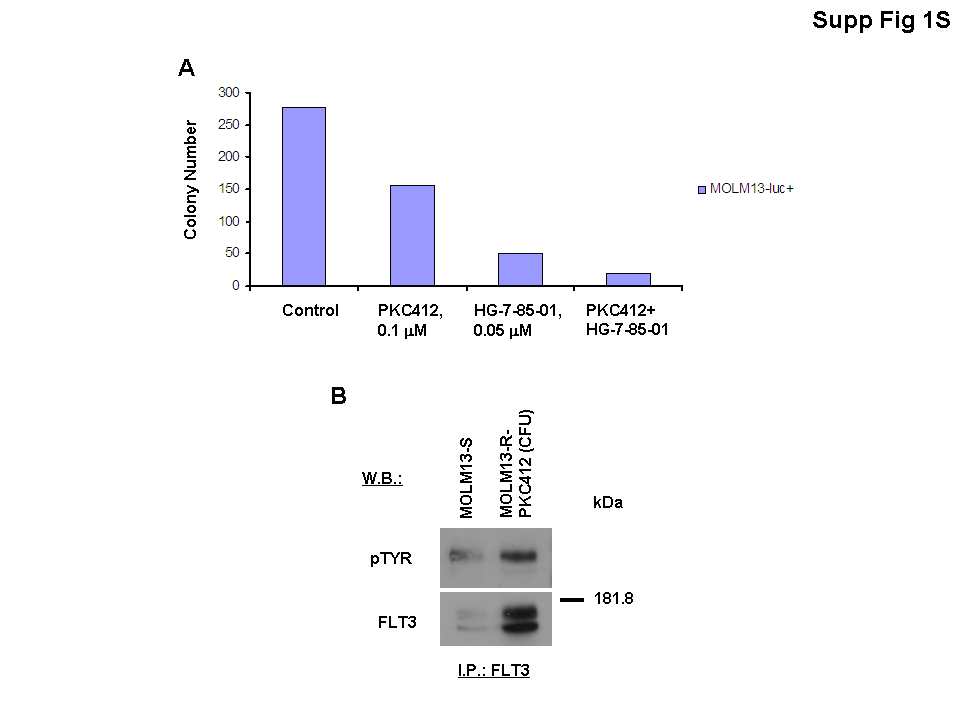

Supplement: Figure S1 — PKC412-resistant cell line (resistant to 100 nM PKC412) developed from Colony Forming Units (CFUs). (A) Colony assays: MOLM13-luc+ cells seeded. 100 cells/0.1 mL in IMDM. 900 uL Metho Cult. Total/well = 1 mL. Stem Cell Technologies “Metho Cult”. Methylcellulose cat # 4230 without cytokines. 9 days between seeding cells and counting. (B) PKC412-resistant cell line (resistant to 100 nM PKC412) developed from Colony Forming Units (CFUs) (MOLM13-R-PKC412 (CFU): pTYR levels and FLT3 protein levels. 12/10/09 FLT3 I.P./western for PKC412-resistant colony (derived from MOLM13-luc+ cells), and compared to wt MOLM13-luc+ cells in culture. For I.P./western, FLT3 Ab (1∶2000). For development of MOLM13-R-PKC412 (CFU) cells, colony assays were initially performed in which 100 MOLM13-luc+ cells/0.1 mL in IMDM were seeded +900 uL “complete” methylcellulose medium containing recombinant cytokines (contents: fetal bovine serum, rh SCF, rh GM-CSF, rh IL-3, Bovine Serum Albumin, methylcellulose in Iscove's MDM, 2-Mercaptoethanol, rh Erythropoietin, L-Glutamine) (MethoCult GFH4434, StemCell Technologies, Inc., Vancouver, BC). The plates also contained PKC412 at the indicated concentrations. The plates were incubated at 37°C in 5% CO2 for >1 week, and then myeloid and erythroid colonies (early progenitors with erythroid and myeloid components: CFU-GM, CFU-E, BFU-E, and CFU-GEMM) were counted on an inverted microscope. There was a total of nine days between seeding cells and counting and drug-resistant colony selection, pooling of colonies, and culture of colonies. (TIF) [file pone.0025351.s001.tif]

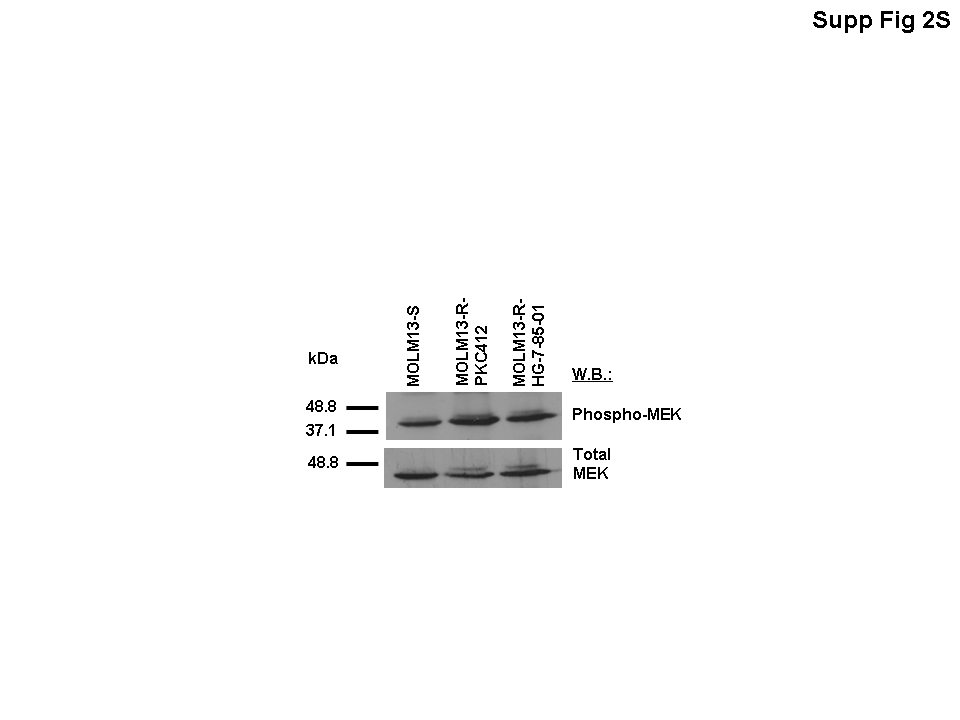

Supplement: Figure S2 — Phospho-MEK expression in MOLM13-R-PKC412 and MOLM13-R-HG-7-85-01 cells. Protein expression was assessed by immunoblotting. (TIF) [file pone.0025351.s002.tif]

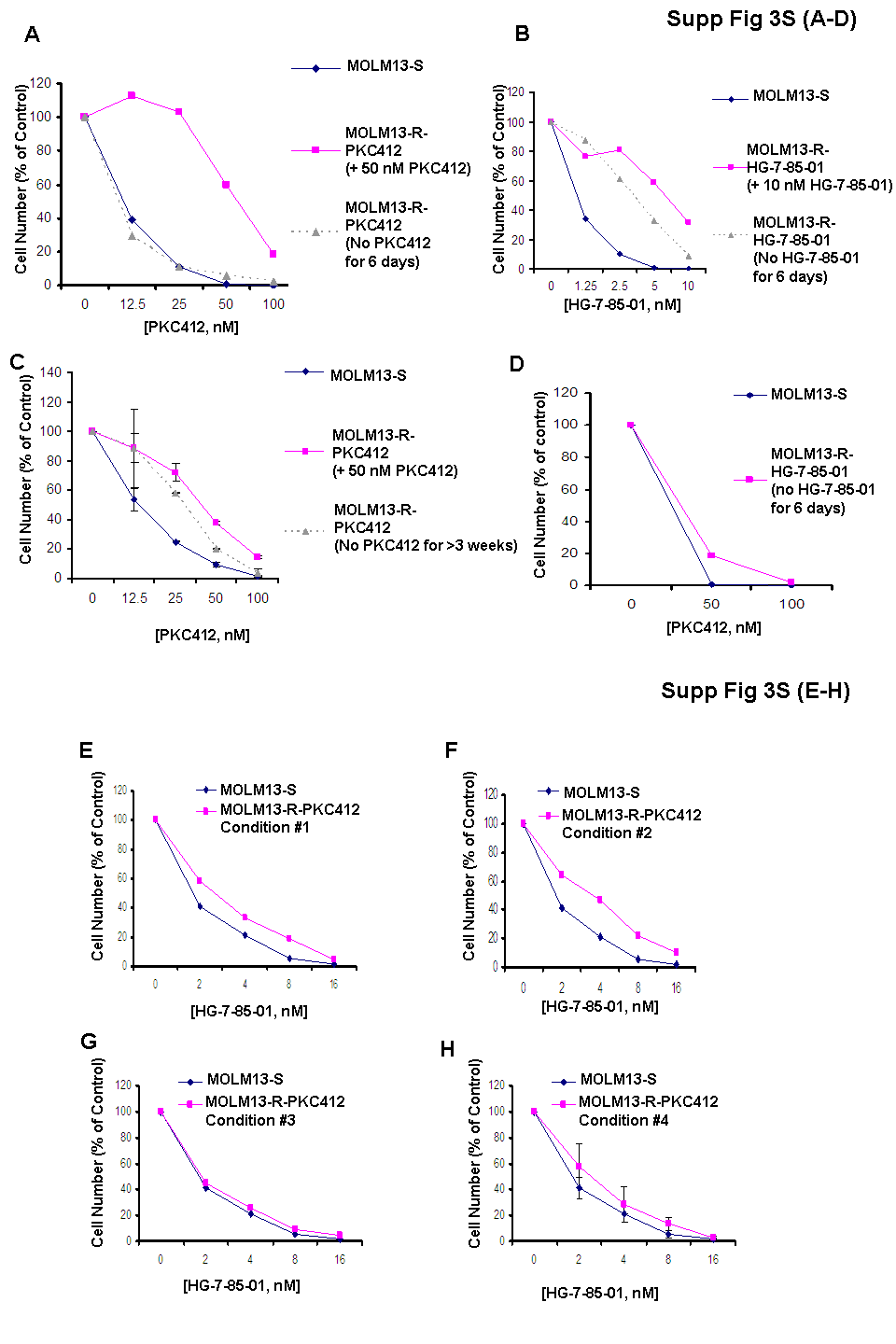

Supplement: Figure S3 — (A–D). Effects of FLT3 inhibitor withdrawal on proliferation of FLT3 inhibitor-resistant cells. (A–B) Effects of short-term drug withdrawal on MOLM13-R-PKC412 and MOLM13-R-HG-7-85-01 cells. (C) Effects of over three week drug withdrawal on MOLM13-R-PKC412 cells. (D) Drug washout experiment: six-day drug withdrawal: effects on proliferation of MOLM13-R-HG-7-85-01 in the presence of PKC412. (E–H). Effects of FLT3 inhibitor withdrawal on proliferation of FLT3 inhibitor-resistant cells. Condition #1: Two-day withdrawal of PKC412 from MOLM13-R-PKC412 cells prior to assay. Condition #2: Two days of PKC412 treatment of MOLM13-R-PKC412, two days of PKC412 withdrawal, three days of PKC412 treatment, and two days of PKC412 withdrawal prior to assay. Condition #3: Five days of PKC412 withdrawal prior to assay. Condition #4: Seven days of PKC412 withdrawal prior to assay. (TIF) [file pone.0025351.s003.tif]

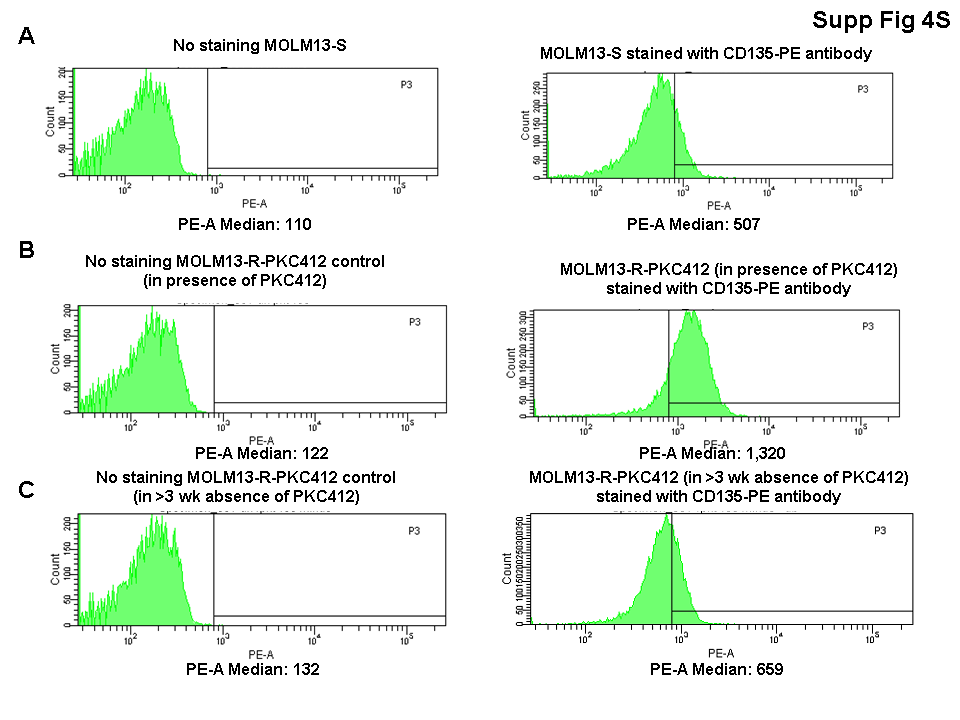

Supplement: Figure S4 — Flow cytometry analyzing surface expression of FLT3 receptor in drug-sensitive cells versus drug-resistant cells cultured in the absence and presence of inhibitor. (TIF) [file pone.0025351.s004.tif]

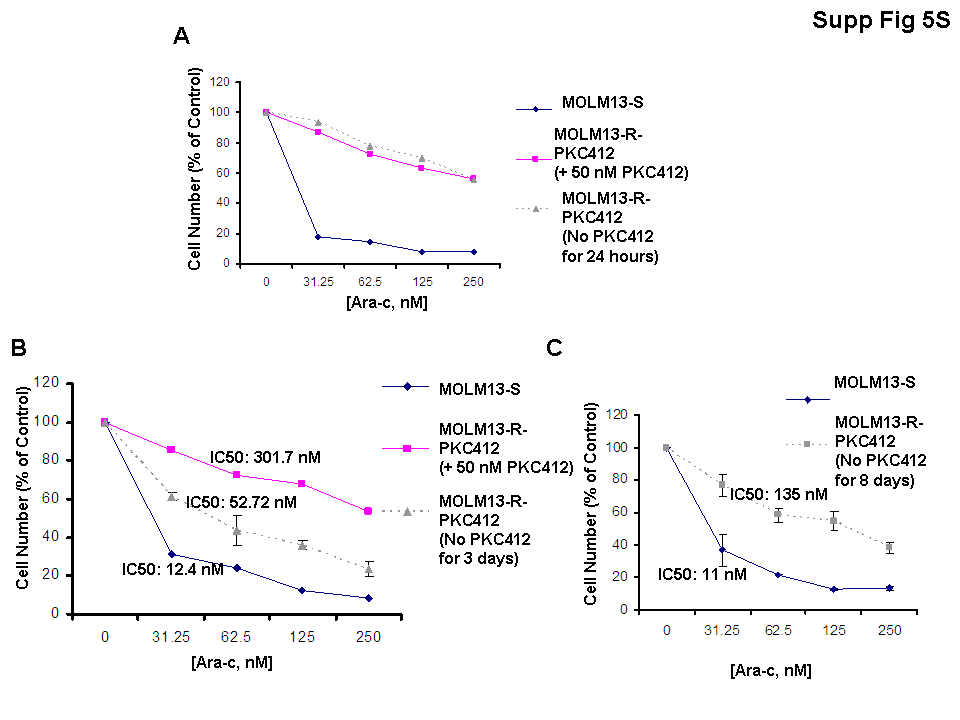

Supplement: Figure S5 — Cross resistance of MOLM13-R-PKC412 cells to standard chemotherapy. (A) Comparison of sensitivity to Ara-c of MOLM13-S and MOLM13-R-PKC412 cells in the continuous presence of PKC412 and following 24 hours of PKC412 withdrawal. (B) Comparison of sensitivity of Ara-c of MOLM13-S and MOLM13-R-PKC412 cells in the continuous presence of PKC412 and following 3-days of PKC412 withdrawal. (C) Comparison of sensitivity of Ara-c of MOLM13-S and MOLM13-R-PKC412 cells in the continuous presence of PKC412 and following 8-days of PKC412 withdrawal. (TIF) [file pone.0025351.s005.tif]

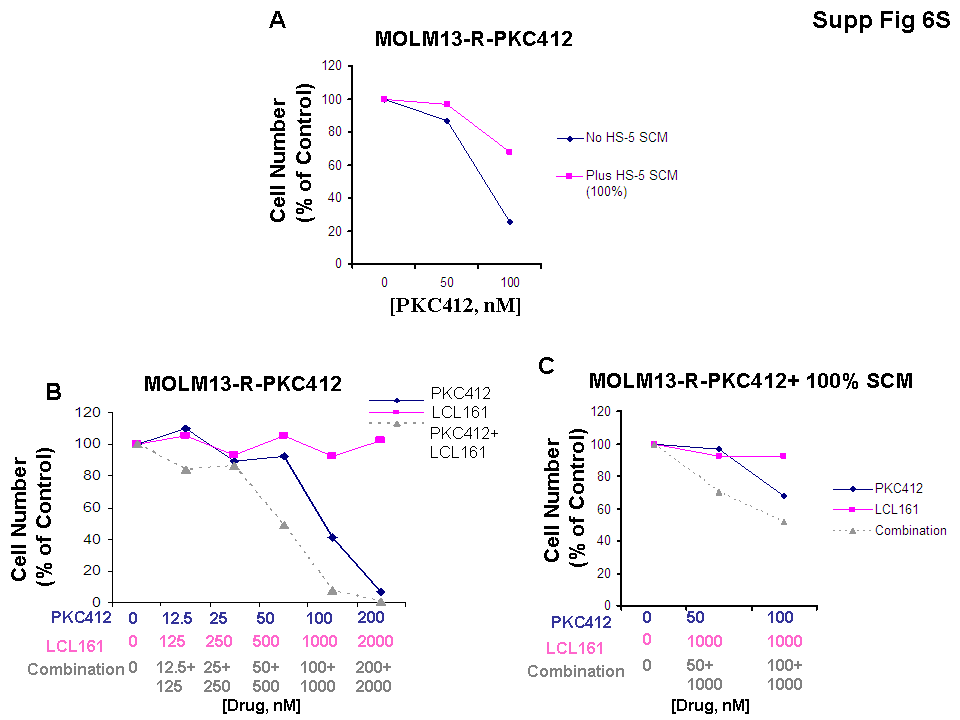

Supplement: Figure S6 — Effects of combination of LCL161 and PKC412 on PKC412-resistant leukemia cells. (A) Stromal-mediated rescue of PKC412-resistant MOLM13-S cells cultured for approximately 3 days in the presence of PKC412. (B) Approximately 3-day treatment of MOLM13-R-PKC412 (cultured in the absence of stromal conditioned media, or SCM) with PKC412, LCL161, or a combination of PKC412 and LCL161. (C) Approximately 3-day treatment of MOLM13-R-PKC412 cells (cultured in the presence of SCM) with PKC412, LCL161, or a combination of PKC412 and LCL161. This study was performed with one fixed concentration (1000 nM) of LCL161. (TIF) [file pone.0025351.s006.tif]
